# Nursing assistant's perceptions of the good work environment in municipal elderly care in Sweden –a focus group study

**DOI:** 10.1108/JHOM-07-2020-0290

**Published:** 2021-04-29

**Authors:** Per-Ola Maneschiöld, Diana Lucaci-Maneschiöld

**Affiliations:** Karlstad University , Karlstad, Sweden; University of Gothenburg , Gothenburg, Sweden

**Keywords:** Sense of coherence, New Public Management, Qualitative research, BIKVA model, Work–life conditions, Nursing home, Nursing assistant

## Abstract

**Purpose:**

The purpose of this paper is to investigate aspects related to difficulty to retain nursing assistants at nursing homes in Sweden related to perceived work environment characteristics.

**Design/methodology/approach:**

To reveal aspects related to difficulty to retain nursing assistants, the paper uses the BIKVA model, sense of coherence and New Public Management (NPM). In total, three focus groups with nursing assistants at three nursing homes are interviewed with corresponding individual interviews with their senior managers and users. The purpose is to analyze the situation from the affected group of nursing assistants. The focus of this study is how nursing assistants discuss related to recruit and retain nursing assistants at nursing homes and elderly care and the response from senior management related to those aspects.

**Findings:**

The main conclusions are that nursing assistants consider their job as meaningful, but limited latitude and direct involvement in managing their daily tasks in a continuous communication with management affect negatively. Furthermore and combined with wage levels, aspects related to scheduling, working hours, shift work, split shifts and understaffing generate a burdensome and stressful environment affecting the possibility to retain staff in a negative direction.

**Originality/value:**

The research uses a new approach utilizing the BIKVA model, sense of coherence and NPM. The study shows that central in retaining nursing assistants at nursing homes relates to aspects such as wages, staffing, shift work and split shifts and continuous communication between nursing assistants and management.

## 1. Introduction

Certified nursing assistants in elderly care, being the direct care staff, are difficult to recruit and retain with high turnover rates ( Decker *et al.* , 2003 ). The high turnover rates and number of vacant positions may compromise continuity of resident care and its quality utilizing stable care teams ( Kash *et al.* , 2006 ). For many reasons, difficulties to retain nursing assistants in elderly care are troubling, including decreased continuity and quality of care, additional costs associated with recruitment and training and adverse effects on both staffing levels and resident outcomes ( Rantz *et al.* , 2004 ; Schnelle *et al.* , 2004 ).

Difficulties to retain nursing assistants can relate to specific conditions, making a nursing home unattractive, e.g. due to poor management and staff-mix affecting staffing levels in long run due to high turnover and difficulties to fill vacancies ( Kash *et al.* , 2006 ). Staff turnover can then serve as an indicator of attractiveness of nursing homes, making it important to focus on work environment characteristics and management initiatives, reducing turnover and ultimately result in higher staffing levels ( Kash *et al.* , 2006 ). Furthermore, policy initiatives should be directed toward improving staff levels and retention where better management capacity and practices as well as work-environment characteristics combined with higher wages can help to improve retention ( Kash *et al.* , 2006 ). Hence, it is of importance to focus on perceived work environment characteristics for nursing assistants to retain in nursing homes as a central concern to maintain and improve the quantity and quality in elderly care services.

Another trend affecting the situation is the decrease, in relative terms, of the workforce and increase of older people in a demography shift, generating an increased demand for elderly care ( Israelsson and Gustavsson, 2004 ). This demography-based increase in demand for nursing assistants is combined with a decline in interest to work at nursing homes, generating an excess demand for nursing assistants. Hence, the excess demand situation generates opportunities for a job carrier in terms of improved employment conditions, such as salary levels and switching between nursing homes as well as between sizes of municipalities, making it more difficult to generate quality utilizing stable care teams. Furthermore, opportunity to switch between nursing homes based on differences in work environment characteristics implies that internal work environment characteristics can be defined as a competitive edge competing for scarce nursing assistants.

As a consequence of difficulties to upgrade supply of elderly care, municipalities in Sweden degrade supply of nursing homes partly due to increased demand for tax income to finance elderly care and partly due to an increasingly difficult recruiting and retaining nursing assistants ( Långtidsutredningen Bilaga 9, 2000 ). Thus, elderly care is assumed to take place, to a larger extent, in the older person's residence with less labor-intensive care services. In spite this degrade in supply of elderly care and nursing homes, the general situation with difficulties to recruit and retain nursing assistants in elderly care persists in Sweden ( Långtidsutredningen Bilaga 9, 2000 ).

Based on this development, it is of significant concern for society to generate an attractive workplace to attract people to elderly care and especially nursing assistants being the largest occupational group. Central in the process to recruit and retain nursing assistants are issues related to increased attractiveness to work in the sector. Examples of work environment characteristics to be improved to increase attractiveness are work environment in general, career ladder possibilities, aspects related to scheduling and working hours, increased latitude within daily duties and improvement in the culture such as continuous communication with colleagues and management as well as salary levels (see, e.g. Szebehely *et al.* , 2017 ; Brannon *et al.* , 2011 ; Hasson and Arnetz, 2008 ).

Analyzing the aspects affecting work environment characteristics and interest to remain in nursing homes is critical to improve nursing home quality. As public policy has to achieve adequate staffing levels and lower turnover rates, it is of interest to analyze aspects related to the interest for nursing assistants to remain employed at nursing homes other than staffing standards to address these problems. This paper will analyze perceived work environment characteristics, other than staffing standards, for nursing assistants to retain in nursing homes and elderly care in Sweden, which will be of central concern for the possibility to maintain and improve the quantity and quality in elderly care services. As a conceptual framework to analyze the nursing assistants' work environment characteristics, the study will use a new approach utilizing the sense of coherence ( Antonovsky, 1987 ). As a complementary theory, New Public Management (NPM) will be used representing an organizational economic constraint to maintain and improve the quantity and quality in elderly care services. Furthermore as part of the new approach, the BIKVA-model with focus group interviews is used were the interviews are based on the aim of the study. The BIKVA-model entitles a specific group the role to question and challenge practice being triggers of learning. The remaining respondents respond to the questions and discussions from the trigger-group ( Krogstrup, 2017 ). In this study, the trigger group is the nursing assistants, defining the aspects to be discussed by the interviewees where remaining respondents are managers as well as users. By using the BIKVA-model, the interviews with managers and users will focus aspects of significance according to the trigger-group of nursing assistants also being the group deciding to remain or not as nursing assistants at nursing homes.

The paper is organized as follows, Section 2 discusses the previous research and theoretical framework, and Section 3 discusses the study. Section 4 analyzes the empirical findings, and Section 5 concludes with a discussion.

## 2. Previous research and theoretical framework

### 2.1 Swedish context and previous research

Nursing assistants' tasks and roles differ between countries, influencing the aspects discussed by interviewees. In the Swedish context, focus areas for nursing assistants at nursing homes include basic and specific individual-centered care, education and skills development and follow-up and revision ( Ehrenberg and Wallin, 2009 ). Individual-centered basic care focuses on the user's nursing needs, including physical and mental needs, and social, cultural and spiritual needs incorporating, e.g. support with personal hygiene and dinning situations, supervise that users have needful and well-balanced activities and rest, observe problems, symptoms and changes in user's health status, conduct, document and evaluate nursing activities and plan nursing activities with colleagues ( Ehrenberg and Wallin, 2009 ). Individual-centered specific care includes, e.g. conduct, plan and assist at medicinal examinations and treatments, document user's response to care activities, inform and educate users and their relatives in a pedagogical motivation concerning the user's habilitation process and give palliative care ( Ehrenberg and Wallin, 2009 ). Rapid knowledge development within health care implies a need for continuous professional training and education, including critical re-evaluation of current care methods, and in dialogue with colleagues and profession, evaluate implementation of new methods, contribute to education and skills development of colleagues and introduction of new employees in a peer system and continuous evaluation of the nursing assistant's own need of professional skills development and education ( Byström, 2013 ). Within follow-up and revision, assignments include, e.g. follow-up and revision of the nursing assistant's own areas of responsibility and communicating outcome in those areas with colleagues ( Ehrenberg and Wallin, 2009 ).

In the previous research, some central results relate to the aim of this paper. The results can be grouped in (1) latitude and meaningfulness, (2) communication and scheduling and (3) workload. The aspects in the above themes affect the perceived work environment characteristics and possibility to retain nursing assistants at nursing homes. This paper will utilize the above themes using the theories sense of coherence and NPM as well as central results in previous research corresponding to the themes, which are presented below.

Josefsson *et al.* (2011) argue that an attractive working environment is central for nursing assistants to remain in elderly care. Central is intellectually stimulating assignments not linked to feelings of guilt, freedom and independence, with the possibility to influence assignments as well as to have appreciating and pleasant co-workers, good relationship with individual users and their relatives and an equitable and understandable manager ( Carlsson *et al.* , 2014 ; Josefsson *et al.* , 2011 ). Furthermore, an attractive working environment incorporates a safe working environment, employment security and secured income with a possibility to increase salary by own initiatives and work efforts ( Carlsson *et al.* , 2014 ; Josefsson *et al.* , 2011 ). To prevent stress, central is a re-balancing of the work-related environment related to content and improving supplementary education and training both formal and in a peer system related to a positive working culture ( Brannon *et al.* , 2011 ; Hasson and Arnetz, 2008 ; Szebehely *et al.* , 2017 ). The culture at the workplace is central in generating an attractive work environment ( Brannon *et al.* , 2011 ).

Szebehely *et al.* (2017) argue that, e.g. terms of employment such as employment type, workload, shift work as well as split shifts affect attractiveness in a work environment. Furthermore, of importance is to focus on aspects that can be improved such as workload and staffing balanced to current care burden with balanced working hours between work and private life as well as re-design of content in assignments to a more flexible, challenging and stimulating job ( Pilemer, 1997 , and Szebehely *et al.* , 2017 ). Of central concern is a latitude within daily duties directly involving the nursing assistants in drafting and managing their daily tasks, possibility to develop in knowledge and ability in a peer support system and a continuous dialogue with management to solve daily work-related situations ( Szebehely *et al.* , 2017 ). Pilemer (1997) discusses the implementation of carrier ladders to improve the knowledge and skill being able to have more advanced assignments as an incentive to remain in elderly care. However, there were, in general, limited resources for such a peer support system, contributing to a stressful working environment ( Carlson *et al.* , 2014 ).

There is a difference in incentives to work in elderly care, where the older person's health status continuously deteriorates with increasing care burden, and to work in, e.g. a hospital, where the general development is to recover the health status affecting incentives to work in elderly care negatively ( Waerness, 1980 ). Wiener *et al.* (2009) argue that, in such a competitive situation, the most central factors are salary and employment security as well as possibility to make a carrier by re-education and applying for other more advanced jobs within elderly care using carrier ladders, as discussed in Pilemer (1997) .

To produce an attractive work environment, NPM and economic efficiency generate a situation in elderly care that is counter-productive in a human capital-intensive production process ( Eliasson-Lappalainen, 2011 ). Thus, NPM in elderly care based on a budget constraint and time- and cost-efficient production will imply a constraint on possibilities to generate an attractive work environment ( Eliasson-Lappalainen, 2011 ). Furthermore, Lauri (2016) argues that NPM produces distance and detachment and thus discourages caring subjects and undermines the cultivation of a relationship between nursing assistants and users. Lauri (2016) argues that the use of NPM will devalue and deter the surfacing of emotions, reducing the amount of time available to meet users with a distancing from users' dependency and needs. More suitable would be to create latitude for the nursing assistants to assess the situation determining the most suitable approach based on their knowledge and experience directly involving them in drafting and managing their daily tasks ( Eliasson-Lappalainen, 2011 ; Szebehely *et al.* , 2017 ).

### 2.2 Theoretical framework

Working as a nursing assistant at nursing homes is characterized by an emotionally and physically burdensome job in a stressful and understaffed environment ( Brannon *et al.* , 2011 ). In such an environment, of central concern for the possibility to maintain and improve quantity and quality in elderly care services are, among other things, perceived work environment characteristics. The aspects affecting those characteristics relate, among other things, to the sense of coherence ( Antonovsky, 1987 ) as an adaptive dispositional orientation that enables coping with adverse experiences such as an emotionally and physically burdensome and stressful job. The sense of coherence is an orientation that expresses the extent to which a person has a feeling of confidence ( Antonovsky, 1987 ). It depends on how stimuli from internal and external surroundings are structured, predictable and explicable through a person's life and how resources are available to a person to meet the demands and how they are regarded as challenges, worthy of investment and engagement ( Antonovsky, 1987 ). A person's life-coping strategies in a burdensome and stressful situation are linked to its context and in relation to the person's experiences of sense of coherence integrating comprehensibility, manageability and meaningfulness ( Antonovsky, 1987 ).

Comprehensibility, the cognitive dimension, is a person's ability to understand what happens around them and how they perceive stimuli confronting them as making cognitive sense and the extent to which events are perceived as making logical sense, that they are ordered, consistent and structured. It relates to a belief that things happen in an orderly and predictable fashion and sense that the person can understand events in his/her life and reasonably predict what will happen in the future ( Silverman *et al.* , 2016 ). The ability to create structure out of chaos makes it easier to understand and cope with stressful situations, in that what one comprehends is easier to manage ( Antonovsky, 1987 ).

Manageability, the instrumental and behavioral dimension, is a person's ability to manage a situation on their own or through significant others in their social network and extent to which a person feels that they can handle the situation ( Antonovsky, 1987 ). It relates to a belief that a person has the skills or ability and the support or resources necessary to take care of the situation, and that the situation is manageable and within the person's control ( Silverman *et al.* , 2016 ). This is equivalent to the extent of resources at a person's disposal necessary to meet the demands posed by various stimuli were coping also requires motivation to solve problem causing stress, willingness to invest effort to solve problems and find a meaning in being able to manage the situation ( Antonovsky, 1987 ).

Meaningfulness, the motivational dimension, is a person's ability to find motivation and emotional meaning in their life situation. It relates to the belief that things in life are interesting and a source of satisfaction, that things are really worth the effort and that there is a good reason or purpose to care about what happens to affect the situation ( Silverman *et al.* , 2016 ). Meaningfulness is about how much a person feels that life makes sense were challenges are seen as challenges, rather than only burdens, worthy of commitment and dedication. Central is that the person needs to have a clear desire to resolve difficulties and willingness to invest energy to get through experiences of stress that have potential to cause distress ( Antonovsky, 1987 ).

Further concepts associated with (1) comprehensibility is predictability, understandability, clarity, insight and structure and rules; (2) manageability is associated with resources, master, knowledge, trust, possibilities, affect, security, control and assets; and (3) meaningfulness is associated with belief in the future, community, engagement, participation, belonging, context and motivation ( Hagström *et al.* , 2000 ).

A person's potential to successfully cope with a situation is assumed to be positively dependent on the level of sense of coherence, i.e. the more a person is able to understand and integrate, i.e. comprehensibility, to handle, i.e. manageability, and to make sense of the situation, i.e. meaningfulness, the higher will be the sense of coherence. Thus, the stronger the sense of coherence, the more able is a person to mediate and ameliorate stresses in situations such as an emotionally and physically burdensome job in a stressful and understaffed environment by influencing coping efforts ( Silverman *et al.* , 2016 ). Hence, a higher degree of sense of coherence will increase the possibility to cope with the burdensome and stressful situation as a nursing assistant at nursing homes and increase the possibility to retain the nursing assistant.

Introduction of NPM in the organization and governing of welfare services imply a possibility to add to the sense of coherence, as NPM is characterized by decentralization ( Hood, 1991 ). A more decentralized control of resources in the organization generates latitude to decide how services should be performed and produced, e.g. by involving nursing assistants in that process. This adds to give good care related to interaction between staff and user as the central aspect ( Lauri, 2016 ). Thus, adding to the sense of coherence, in turn increasing the possibility to cope with the burdensome and stressful situation as a nursing assistant at a nursing home.

However, NPM assumes that management in the public sector can be based on increased productivity by efficient use of resources ( Baldersheim and Rose, 2010 ). This implies increased focus on evaluation, documentation and measuring accomplishments and efficiency of the organization ( Hood, 1991 ). Central is a command and control mode of functioning by identifying and setting targets and monitoring performance, with a strong focus on financial control to increase efficiency. Furthermore, NPM includes an auditing function at both the financial and professional levels using transparent means to review performances, setting benchmarks, using protocols to ameliorate professional behavior with greater customer orientation and responsiveness to user demands ( Hood, 1991 ). As a result, this can decrease the sense of coherence, focusing on cost-efficient production, budget awareness and lack of resources limiting aspects such as predictability and understandability of governing with negative impact on engagement and motivation. Furthermore, NPM produces distance and detachment and thus discourages caring subjects and undermines the cultivation of a relationship between the nursing assistant and user ( Lauri, 2016 ). An outcome is that it devalues and deters the surfacing of emotions, reducing the amount of time available to meet users with distancing from users' dependency and needs ( Lauri, 2016 ).

As a complementary theory to analyze the empirical material and implications of perceived work environment characteristics, NPM will be used representing an economic constraint on the quantity and quality in elderly care services, with implication for the magnitude of sense of coherence. Economic resources as a constraint can entail implications for sense of coherence and its magnitude and development within an organization related to economic resources as a resource to develop, e.g. manageability and meaningfulness with a belief in the future of elderly care services of highest standard.

## 3. The study

This study was based on qualitative semi-structured interviews in focus groups as a method to collect data to be able to illuminate nursing assistants' experiences from different perspectives. Based on the research aim, a conventional qualitative content analysis was used as suitable method of analysis ( Hsieh and Shannon, 2005 ). Conventional qualitative content analysis can be used to interpret the content of data through a systematic process and aims to describe the nursing assistants' experiences from different perspectives ( Hsieh and Shannon, 2005 ). The study used the theory of sense of coherence ( Antonovsky, 1987 ) as an inspiration to both the interview guide and analysis of the empirical material. As a complementary theory, NPM was used representing an organizational economic constraint to maintain and improve the quantity and quality in elderly care services.

The recruitment process and interviews with participants took place during autumn 2019 at three residential homes in Sweden whereof two in a smaller municipality and one in a larger urban municipality. The purposive sample consisted of 11 nursing assistants, four managers and four users. Managers and users were from the same residential homes as the nursing assistants being the nursing assistants' managers or users at the department were the nursing assistants work. All respondents were female. The nursing assistants were between 24 and 64 years of age, the managers between 30 and 65 years of age and the users between 78 and 90 years of age. Average relevant work experience for the nursing assistants were 17 and 15 years for the managers, excluding years as a nursing assistant in elderly care. At total, 19 persons were included in the study, as with that number, the authors assessed that data saturation was achieved ( Burmeister and Aitken, 2012 ; Dibley, 2011 ; Fusch and Ness, 2015 ). None of the respondents declined participation.

The sample should be seen as a case study instead of a generalized study. However, the study is still enabling an analysis to provide an understanding of the respondents' experiences of their working lives when discussing the topic related to difficulties to recruit and retain certified nursing assistants in nursing homes ( Meyer, 2015 ). Furthermore, the results in the study are still transferable to other cases, in that the respondents represent a representative sample of staff and users at an average nursing home.

To collect data, the BIKVA model was used, giving a specific group the role to question and challenge practice being triggers of learning were remaining respondents respond to questions and discussions from the trigger group ( Krogstrup, 2017 ). In this study, the trigger group are the nursing assistants defining the area of questions and discussions for managers and users to respond too. During the focus group interviews with the nursing assistants, the central aspects and concerns based on the aim of the study generated the content in the following individual interviews with the managers at each nursing home and with users in line with the BIKVA model ( Krogstrup, 2017 ). This focuses the discussion on the aspects of importance to the group deciding to remain or not as nursing assistant at nursing homes.

Three focus group interviews, one at each nursing home, including between four to six nursing assistants, were conducted. The interviews with the managers were conducted as individual interviews. Each interview, both focus groups interviews and individual interviews, were recorded by Dictaphone and lasted up to 120 min and were carried out in a separate room at each nursing home. Both authors conducted the interviews were one author moderated the interviews, with a focus on helping the respondents to focus on the topic, while the other author assisted by asking probing questions and keeping notes ( Wibeck, 2011 ). As support helping to focus discussions, an interview guide was used based on the aim of the study, with foundation in the sense of coherence ( Antonovsky, 1987 ) and NPM. Questions were open questions, and interviews were later transcribed, but transcriptions were not returned to the respondents for comments. However, for each interview, the authors secured the possibility to return to the respondents if further issues needed to be clarified.

Interviews continued until the researchers experienced that no new information emerged, giving respondents the time needed to discuss and express their thoughts. After the third focus group interview at the third nursing home with corresponding individual interviews with management and users, the researchers experienced that no new information emerged, and no further interviews were needed at other nursing homes for data saturation.

To perform data analysis, a deductive thematic analysis were used to analyze the material with aim to identify, analyze and find patterns or themes ( Braun and Clarke, 2006 ). The latent thematic analysis was inspired by the sense of coherence ( Antonovsky, 1987 ) and NPM as well as previous research. First, interviews were transcribed verbatim, and the text was read several times to obtain an overall view of the empirical material. Second, the material was thematically structured inspired by sense of coherence and NPM. As the analysis proceeded, labels for codes emerged that were reflective of more than one key concept, and together, the codes resulted in the initial coding scheme. Each interview was analyzed separately, and quotes from the interview were organized in the thematic structure. Third, the thematically structured material was analyzed focusing on the similarities and differences, with a focus on identifying codes that captured the key concepts and thoughts. Fourth, themes were constructed based on this analysis and the aim of the study, with foundation in sense of coherence and NPM. A consensus was reached and resulted in three themes represented by (1) latitude and meaningfulness, (2) communication and scheduling and (3) workload. Quotes were selected from the empirical material to illustrate the analysis.

While conducting data collection and data analysis, the study followed the principles of the Helsinki Declaration ( World Medical Association, 2013 ) and the Swedish ethical guidelines ( Gustafsson *et al.* , 2005 ). All participation was voluntarily, information about the study was given orally and in writing prior to the study and informed consent obtained. Participants were informed that they could withdraw from the study at any time without explanation, and that data were anonymized and kept inaccessible to anyone other than the researchers.

## 4. Findings

### 4.1 Latitude and meaningfulness

The most important aspect to manage stressful situations is the sense of coherence ( Hagström *et al.* , 2000 ). The nursing assistants at all nursing homes in the study consider their job as meaningful, and that belonging to a team of colleagues creates a significant feeling of safety in the job situation and workplace. One nursing assistant said:

When I leave my shift, insignificant issues says it all generating meaningfulness being appreciated for who you are and what you do feeling gratitude, comfort and safety at work in return from users and colleagues. That makes me to enjoy and motivates me to continue even in hardships.

This connects to meaningfulness being the motivational dimension. It implies the ability to find motivation and emotional meaning in the life situation and how much the person feels that life makes sense and not only being a burden. The nursing assistants express a clear desire to resolve difficulties and willingness to invest energy to get through situations of stress with potential to cause distress. Antonovsky (1987) argues, central in meaningfulness is an emotional content and meaning in a life situation to improve the well-being expressed by the nursing assistants. Further concepts associated with meaningfulness include the belief in the future, community, engagement, participation, belonging, context and motivation, which are concepts linked to what the nursing assistant expresses as motivating aspects ( Hagström *et al.* , 2000 ). This is a significant aspect among staff at nursing homes to thrive and enjoy work with a good relationship with users and other colleagues ( Carlson *et al.* , 2014 ). Furthermore, it is of importance to work with colleagues and managers, creating a pleasant environment to generate motivation in a stressful job situation ( Josefsson *et al.* , 2011 ).

The insight of meaningfulness can emerge with a significant time lag relative to the specific situation itself, implying a time perspective related to meaningfulness. This aspect can generate meaningfulness much later, creating motivation in the long run based on knowing that what I do is valued even if no token of appreciation is expressed immediately. This adds to the knowledge base, especially for long-term staff, adding to the quality and satisfaction at the job finding motivation and emotional meaning in the stressful job situation. One nursing assistant expresses the aspects as:

Relatives to a user that passed away always had comments on how we cared for the user. After some time, they came and expressed how satisfied they were with all care. I just thought they were disappointed all the time. Those moments you never forget meaning a lot in periods without positive response.

This highlights that experiences of appreciation with a significant time lag can enhance and amplify the aspect of meaningfulness and sense of coherence as a motivational aspect to better manage stressful job situations, thus, adding a time perspective to meaningfulness and sense of coherence contributing to the importance to retain nursing assistants in a long-term perspective at nursing homes.

Increased latitude within daily duties directly involving nursing assistants in drafting and managing their daily tasks can contribute to the sense of coherence, linking to comprehensibility and manageability experiencing a predictable and controllable future. The nursing assistants have different experiences with a more positive experience from nursing assistants with a greater latitude and direct involvement. This positive experience is expressed by a nursing assistant as:

…manager delegates to us to decide how to solve a situation with possibility to come back if needed. There is a daily continuous dialog with the manager and we have our guidelines assisting. This generates a latitude in drafting and managing daily tasks based on guidelines generating predictability and satisfaction.

At one of the other nursing homes, latitude and direct involvement are more limited. The nursing assistants' experience from this nursing home is less positive, affecting their job situation in a negative direction. One nursing assistant said:

It would be much better if we can decide on scheduling and staffing but we are not. The manager decides and there is difficult to establish a dialog. We are not even allowed to switch shifts if needed to generate a better balance between job and private life. That is not satisfying.

However, this limited possibility of latitude and direct involvement is not shared by their manager saying:

In the end, I am deciding but it is of importance to get staff directly involved in the decision making which I believe they are to a significant extent in line with our routines.

At this nursing home, the manager's opinion is that staff is involved according to routines, but that the nursing assistants experience a difficulty to establish a dialogue even related to aspects such as to generate a better balance between job and private life. Thus, there is a miss-communication and gap between the nursing assistants and their manager concerning latitude and involvement affecting the nursing assistants' experienced job satisfaction in a negative direction. Latitude and direct involvement with continuous communication with management is of importance for the nursing assistants' experienced job satisfaction, motivation and development in job skills ( Carlson *et al.* , 2014 ; Josefsson *et al.* , 2011 ; Szebehely *et al.* , 2017 ).

### 4.2 Communication and scheduling

Central in retaining nursing assistants is a more involving and allowing management, with continuous communication as part of a positive culture ( Brannon *et al.* , 2011 ). Continuous communication with management to solve daily work-related situations as well as knowledge development in a mentorship in a peer support system with colleagues and management is central for improving the work environment as a cultural shift ( Szebehely *et al.* , 2017 ; Josefsson *et al.* , 2011 and Brannon *et al.* , 2011 ). Furthermore, central in improving the work environment is appreciating and pleasant colleagues and an equitable and understandable manager ( Josefsson *et al.* , 2011 ). According to the nursing assistants, communication with management is inadequate, with narrow possibilities to solve daily work-related situations in turn affecting communication with colleagues and work-related atmosphere negatively. One nursing assistant said:

Communication with management on a daily basis is inadequate in turn affecting work-related atmosphere and communication between the nursing assistants. This implies difficulties to solve daily work-related situations and continuous communication between colleagues in a knowledge transfer affecting work-environment negatively.

The managers agree that continuous communication is central, but in proper forums such as formal section meetings or internal digital systems, instead of a spontaneous informal communication. One manager said:

Continuous communication is vital on a daily basis being part of an active leadership and, in my opinion, a natural aspect of this section's leadership. However, it need to be formally addressed in right forum such as section meetings where spontaneous communication generate a possibility that the issue will not be properly solved.

There is a dual opinion about the quantity and quality in continuous communication between the nursing assistants and management. In contrast to management, the nursing assistants' opinion is that it is not optimal for solving daily work-related situations and management refer to communication in proper forums. Hence, there is a gap in assessment of quantity and quality in communication between the nursing assistants and managers. The nursing assistants assess quantity and quality in communication with managers as not optimal affecting work-environment negatively.

Aspects related to scheduling, working hours, shift work and split shifts combined with salary are central in retaining nursing assistants where working hours should be better balanced, taking into consideration the relation between work and private life ( Szebehely *et al.* , 2017 ; Carlson *et al.* , 2014 ; Wiener *et al.* , 2009 ). One nursing assistant said:

If you would like to retain and recruit you need to reconsider scheduling, working-hours and split shifts. Especially if you combine with the low salary, it is not. A better balance between working-hours and private life is needed.

The managers agree on the problematic aspects related to scheduling, working hours and split shifts. They argue that nursing homes are not the only sector with this specific situation being part of a sector working in shifts as a labor market characteristic. However, the management level disagrees that it is a low-wage occupation, and one manager said:

Concerning scheduling, working-hours and split shifts, it is a problem but we are not the only sector with that situation. I disagree with that salary is low. If we compare with the University hospital, we have significantly higher and competitive salaries but perhaps not the same status with users not improving in health-status.

The manager argues for that there is an incentive to work at the university hospital where the general situation for patients is improvement and recovery in health status ( Waerness, 1980 ). Thus, there is a need to have a significantly higher salary at nursing homes to balance incentives and competitiveness at the labor market in line with Carlsson *et al.* (2014) and Wiener *et al.* (2009) . However, the nursing assistants disagree with a different opinion that wages are low and unattractive.

### 4.3 Workload

Workload for nursing assistants in nursing homes is characterized by an emotionally and physically burdensome job in a stressful and understaffed environment, affecting the possibility to retain staff in a negative direction ( Brannon *et al.* , 2011 ; Hasson and Arnetz, 2008 ; Szebehely *et al.* , 2017 ). In line with this argument, the nursing assistants unanimously agree on the burdensome workload and its negative effect. Furthermore, they also mean that workload has increased because the users are older and more care demanding when they move to nursing homes, but that staffing is unchanged. One nursing assistant said:

We are not optimally staffed with even less staffing during evenings and weekends. The care-demand is increasing all the time as users coming are more care-demanding being older and sicker but staffing is unchanged to meet higher demands. That makes me feel insufficient.

One possibility to mitigate the increasing care demand is supplementary education and training, mentorship in a peer system and knowledge development for nursing assistants to be able to appropriately handle more complex care situations ( Brannon *et al.* , 2011 ; Carlsson *et al.* , 2014 ; Pilemer, 1997 ). However, there is very limited time and allowance in the budget for formal and informal education and training in a peer system with colleagues. The argument is an economic constraint based on the budget as well as a time constraint related to understaffing based on the care burden. One nursing assistant said:

Stress is always present with understaffing and no possibilities to re-educate or develop. Always the most urgent….Upon supplementary education and training, manager says that there is very limited resources available but training in a peer system is within the daily work-assignments. But time is never there so it is not satisfying.

Hence, the possibility to be able to handle the more time-demanding and complex care situations by supplementary education and training of staff to mitigate understaffing is not sufficiently present. This development is confirmed by a manager saying:

…development in society with continuous increase of older people and increasing average lifespan generating a continuous increase in elderly care-demand….budget does not allow increased staffing so what should be down-graded?

Workload and staffing should be balanced to current care burden to generate a positive work environment ( Szebehely *et al.* , 2017 ). However, as mentioned by the manager, the budget is a central constraint. In line with NPM, this does not allow sufficient staffing or sufficient supplementary training arguing for a cost-efficient production of care services.

A further aspect is the continuous increase in the administrative burden such as documentation linked to care-giving assignments, but still not care-giving. With an understaffed situation, this implies an increased burden for the nursing assistants, decreasing time available for care-giving. One nursing assistant said:

Administrative work and documentation increases with hardly no time to spend with users but just basic care-giving. This situation is continuously worsening and we are supposed to do that on top of everything else. There is too much to do and not enough staffing. It is very stressful and makes me downhearted.

The users confirm the stressful situation interpreted as understaffing where one user said:

They never have time with anything especially doing it calm and gentle. Staffing is not enough being too few and always stressed.

The manager confirms this general development, but interpretation focuses on sufficient time needed for different assignments linked to planning and staffing. The manager said:

Administrative assignments and documentation has increased partly related to adjustments in legislation. However, it is valuable doing this being able to see what nursing assistants are doing which we did not fully know before. Now we know making it easier to assign optimal amount of time and sufficient staffing using resources more efficient.

The combined interpretation is that workload has increased but when nursing assistants and users argue for understaffing managers argue for optimal staffing and efficient use of limited resources in line with NPM. Thus, the emotionally and physically burdensome job with a stressful and understaffed environment is partly because of economic constraints and argument for economic efficiency in line with NPM. Efficiency in line with NPM in the public sector such as nursing homes is counter-productive due to limited possibility to increase productivity in a human capital-intense production ( Eliasson-Lappalainen, 2011 ). More suitable would be to create latitude for nursing assistants to assess the situation determining the most suitable approach in daily duties based on their knowledge and experience directly involving them in drafting and managing their daily tasks ( Brannon *et al.* , 2011 ; Carlson *et al.* , 2014 ; Eliasson-Lappalainen, 2011 ; Josefsson *et al.* , 2011 ; Szebehely *et al.* , 2017 ).

As a consequence of an increased workload and chronic understaffing linked to that users are older and more care demanding, the nursing assistants experience a very stressful work environment, with a continuous lack of time with the sense of being insufficient in care-giving. This generates an ethical dilemma for the nursing assistants expressed by a nursing assistant as:

Stress is everywhere. You are somewhere else in your mind not present as it should be to take time needed for the most basic. Other users continuously call on your attention but we are so understaffed and when I finally come, users are not at all too happy. It is very stressful feeling insufficient and always trying to be time-efficient making work emotionally exhausting. It generates an emotional conflict with no possibility to take proper care of users.

However, this is not shared by the manager saying:

Why stressing and to what? My opinion is that it is the nursing assistant herself putting this stress upon herself to make it in “time” when we do not have such time-frames when to be done.

There is an obvious dual opinion about if there is a stressful work environment and its origin being continuous understaffing based on efficiency in line with NPM or generated by the nursing assistants themselves. As a response to the dual opinion, a user commented:

…nursing assistants always says … “I am coming” … but they never do not having time even with basics. That is not good care but they are understaffed and stressed and management does nothing to solve it.

Hence, the user confirms the nursing assistants' opinion concerning the time constraint and understaffed situation. For a more attractive working environment, of importance is an active and continuous communication between nursing assistants and management directly involving nursing assistants in drafting and managing their daily tasks in a continuous dialogue with management to solve daily work-related situations ( Szebehely *et al.* , 2017 ). Furthermore, Szebehely *et al.* (2017) argue that workload and staffing should be balanced to current care burden, but the situation contradicts to the recommendations in, e.g. Szebehely *et al.* (2017) .

Workload, general work environment characteristics and general excess demand situation for nursing assistants generate opportunities for a job carrier in terms of improved employment conditions, such as salary levels and switching between nursing homes as well as between sizes of municipalities and to, e.g. university hospitals. Especially for younger nursing assistants, there is an incentive to move from smaller to larger urban municipalities, as part of the urbanization trend, but also an incentive to shift to the university hospital. One young nursing assistant in the smaller municipality said:

….an interest to move to a larger urban municipality with more possibilities in life but also to increase salary. It also generates a possibility to shift to the University hospital with better possibilities concerning more interesting assignments, development by supplementary education and training and to increase salary.

Thus, there is an incentive, especially by younger nursing assistants, to shift from smaller to larger urban municipalities as well as to shift to the university hospital. Hence, it is of greater importance for smaller municipalities to improve working characteristics as well as salary to be competitive to retain nursing assistants at the nursing home, but also in the municipality. One manager in the smaller municipality said:

We are trying to retain nursing assistants making the overall situation attractive both including salary-levels and work-environment characteristics. However, the budget implies limitations still being able to work with aspects such as scheduling and peer education and training.

In the larger urban municipality, the manager argues for competitive salaries but lack in status relative to the university hospital as an argument for the shift from nursing homes to the university hospital:

If we compare with the University hospital, we have significantly higher and competitive salaries but perhaps not same status.

Hence, there is a difference between the small and larger urban municipality with incentives to shift to a larger urban municipality, especially for young nursing assistants. The larger urban municipality faces instead incentives to shift to the university hospital, thus, implying, to some degree, different strategies to retain nursing assistants. The smaller municipality faces a shift from the municipality to a larger urban municipality, but the larger urban municipality faces a shift to the university hospital within the municipality, implying a possibility for a shift back to a nursing home within the municipality.

## 5. Discussion and conclusion

As public policy has to achieve adequate staffing levels and lower turnover rates, it is of interest to analyze aspects, other than staffing standards, related to the interest for nursing assistants to remain employed at nursing homes. This study sets out to reveal aspects for nursing assistants to retain in nursing homes in Sweden of a central concern for the possibility to maintain and improve the quantity and quality in elderly care services.

The nursing assistants consider their job as meaningful, being the motivational dimension in sense of coherence. They express a clear desire to resolve difficulties and willingness to invest energy to get through situations of stress with potential to cause distress being central in meaningfulness ( Antonovsky, 1987 ). The insight of meaningfulness can emerge with a significant time lag, implying a time perspective, adding to the importance to retain staff in a log-term perspective. Furthermore, nursing assistants have different experiences of latitude and direct involvement in drafting and managing their daily tasks with a more positive experience with greater latitude and direct involvement. Latitude and direct involvement with continuous communication with management are of importance for the nursing assistants' experienced job satisfaction, motivation and development in their job skills ( Carlsson *et al.* , 2014 ; Josefsson *et al.* , 2011 ; Szebehely *et al.* , 2017 ).

Central in retaining nursing assistants is a more involving and allowing management with continuous communication as part of a positive culture ( Szebehely *et al.* , 2017 ; Josefsson *et al.* , 2011 , and Brannon *et al.* , 2011 ). According to the nursing assistants, communication with management is inadequate, with a narrow possibility to solve daily work-related situations, in turn affecting communication with colleagues and work-related atmosphere negatively. Hence, a management–communication gap exists in that the nursing assistants assess the quantity and quality in communication as not optimal, affecting the work environment negatively. Furthermore, aspects related to scheduling, working hours, shift work and split shifts combined with salary are central in retaining nursing assistants ( Szebehely *et al.* , 2017 ; Carlsson *et al.* , 2011 ; Wiener *et al.* , 2009 ). The management level agrees on the problematic aspects related to scheduling, working hours and split shifts but argues for a specific labor market characteristic while disagreeing that it is a low-wage occupation.

The workload is burdensome in a stressful and understaffed environment, affecting the possibility to retain staff in a negative direction. Furthermore, the nursing assistants mean that workload has increased because of more care-demanding users but with unchanged staffing and limited possibilities to supplementary education and training to mitigate increased care demand. The management argues for optimal staffing and efficient use of limited resources in line with NPM where stress is generated by the nursing assistants themselves. Hence, there is a dual opinion about if the stressful work environment is due to understaffing based on efficiency in line with NPM or generated by the nursing assistants themselves where the nursing assistants' opinion is confirmed by the users. Furthermore, there is an incentive for a shift from smaller to larger urban municipalities, especially for younger nursing assistants, and an incentive to work at a university hospital relative to nursing homes.

Instead and in line with NPM and efficiency, the result generates an unattractive working environment with a chronic understaffing with limited time for re-education and possibilities to have a latitude within the daily duties in an active communication between nursing assistants and management, generating an ethical dilemma of not doing enough.
